# Assessment of regional left ventricular myocardial strain in patients with left anterior descending coronary stenosis using computed tomography feature tracking

**DOI:** 10.1186/s12872-020-01644-5

**Published:** 2020-08-08

**Authors:** Xiaoyu Han, Yukun Cao, Zhiguo Ju, Jia Liu, Na Li, Yumin Li, Tong Liu, Heshui Shi, Jin Gu

**Affiliations:** 1 Department of Radiology, Union Hospital, Tongji Medical College, Huazhong University of Science and Technology, Wuhan, 430022 China; 2 Hubei Province Key Laboratory of Molecular Imaging, Wuhan, 430022 China; 3 College of Medical Imaging, Shanghai University of Medicine & Health Science, Shanghai, China

**Keywords:** Computed tomography angiography, Coronary artery disease, Strain, Ventricular function

## Abstract

**Background:**

Computed tomography feature tracking (CT-FT) has emerged as a valuable method for the assessment of cardiac function. However, no studies have investigated the usefulness of CT-derived assessments of left ventricular (LV) strain in coronary artery disease (CAD). Our aim was to evaluate regional LV systolic deformation in patients with left anterior descending coronary artery (LAD) stenosis using CT-FT.

**Methods:**

Seventy-six patients with LAD stenosis were enrolled. The patients were divided into four groups according to the percentage of LAD stenosis: ≤25% was defined as group I (24 patients), 26 to 49% as group II (17 patients), 50 to 74% as group III (21 patients), and ≥ 75% as group IV (14 patients). Thirty-two sex- and age-matched healthy subjects were included as controls.

**Results:**

No intergroup differences were found between groups I-IV and the controls in terms of the left ventricular ejection fraction, end-diastolic volume and end-systolic volume. However, the longitudinal strain (LS) of the LAD territory was significantly reduced in groups I-IV compared with the controls (− 20.8, − 18.6%, − 18.6%, and − 17.0% vs − 23.7%, respectively). The circumferential strain (CS) of the LAD territory was significantly reduced in groups III and IV compared with the controls and groups I and II (− 22.4% and − 22.1% vs − 25.4, − 24.1%, and − 25.3%, respectively). Compared with the non-LAD territory, the LAD territory in groups II-IV showed significantly increased LS (− 18.6% vs − 21.9%, *p* = 0.07; − 18.6% vs − 21.9%, *p* = 0.024; − 17.5% vs − 20%, *p* = 0.032, respectively). The severity of LAD stenosis was positively correlated with the LS of the LAD territory (*r* = 0.438, *p =* 0.002).

**Conclusion:**

CT-FT can detect decreasing LV systolic function in patients with LAD stenosis. LV regional systolic deformation of the LAD territory was reduced with increasing LAD stenosis severity.

## Background

In the past decade, the burden of ischemic cardiovascular disease has risen to the leading cause of morbidity and mortality worldwide [1], notably due to coronary artery disease (CAD) [2]. The degree of coronary stenosis is considered the underlying cause of ischemia. The most common vessel involved in CAD is the left anterior descending artery (LAD), which is also the most common infarct-related artery in acute myocardial infarction (MI) [3]. However, the majority of patients with significant LAD stenosis without a history of MI frequently have normal resting electrocardiograms (ECG) and echocardiogram [4, 5]. Although coronary angiography (CAG) is the gold standard for coronary stenosis diagnosis, computed tomography angiography (CTA) is the tool most widely used to visualize the coronary artery because of its noninvasiveness, high degree of temporal resolution and excellent measurement reproducibility [6, 7].

Cardiac strain imaging is a quantitative tool used in the early evaluation of regional systolic cardiac function using echocardiography and cardiac magnetic resonance (CMR) [8, 9]. CTA-derived assessment of strain has emerged as a valuable method for the assessment of cardiac function, and it shows close correlations with both echocardiography and CMR [10–13]. The usefulness of CT-derived assessments of left ventricular strain in severe aortic stenosis [11], aortic valve stenosis [14], adult congenital heart disease [15] and MI [16] has been reported. However, to the best of our knowledge, there have been no studies on CAD.

Consequently, we aimed to evaluate the feasibility of CTA-derived assessments of strain for regional left ventricular (LV) systolic deformation in patients with different degrees of isolated LAD stenosis.

## Methods

### Study population

A total of 119 patients with suspected coronary heart disease were prospectively screened by the multidisciplinary thoracic oncology group at the Union Hospital of Tongji Medical College. Among them, 87 patients were diagnosed with LAD stenosis. Then, 32 sex- and age-matched normal subjects were included as controls.

The inclusion criteria for the LAD stenosis patients were (1) patients diagnosed with LAD stenosis (not including stenosis of the left circumflex coronary artery or right coronary artery); (2) clinically confirmed subjects aged between 30 and 80 years; and (3) no wall motion disorder on echocardiography. The exclusion criteria were (1) a history of MI or Q-waves on ECG; (2) known MI, congestive heart disease, heart valve disease, or structural heart disease; (3) atrial fibrillation; and (4) arrhythmias. All individuals were examined with a cardiac CT scan.

Data regarding demographics and medical comorbidities were collected prospectively before the cardiac CT scan. Nonsmoking was defined as lifetime exposure to fewer than 100 cigarettes, and the remaining patients were categorized as ever-smokers. Patients who consumed alcohol were defined as having a positive alcohol consumption history, and the others were classified as never consumers. Written informed consent was obtained from all participants.

### CT scanning protocol and postprocessing

CT examinations were performed using a DSCT scanner (Somatom Definition, Siemens AG, Medical Solutions, Forchheim, Germany), and the CTCA scanning parameters were as follows: detector collimation 32 × 0.6 mm, slice acquisition 64 × 0.6 mm by means of a z-flying focal spot, gantry rotation time 330 ms, pitch of 0.2–0.39 adapted to the heart rate, reference tube current-time product 400 mAs, and tube voltage 120 kV.

Prior to the examination, patients with a heart rate higher than 75 bpm were treated with a β-blocking agent (metoprolol, 25–50 mg, AstraZeneca AB, Sweden) to control the heart rate below 75 bpm, and all patients were instructed regarding breath holding to minimize artifacts during examination. Nitroglycerin spray (0.5 mg, Shandong Province, China) was used sublingually 5 min before the examination to dilate the coronary arteries. The CTCA scan was started by continuously injecting a total of 70 ml of iopamidol (400 mgI/ml; Bayer Schering Pharma, Berlin, Germany) followed by 40 ml of saline solution into an antecubital vein via an 18-gauge catheter (injection rate 4 ml/s) by an automatic injector. Bolus tracking was used to start the scan with a tracking area set at the root of the descending aorta. The image acquisition started 5 s after the signal attenuation reached the predefined threshold of 100 Hounsfield units. A retrospective gating technique was used to synchronize the data reconstruction with the ECG signal. We reconstructed 20 phases in 5% steps of the R-R interval within the full window. The data constructive section thickness was 0.75 mm, the increment was 0.5 mm, and the reconstruction kernel was B26f heart view smooth without using the iterative reconstruction technique. The image postprocessing methods on the workstation included 3D volume rendering (VR), multiplanar reconstruction (MPR), curved planar reconstruction (CPR), and maximum intensity projection (MIP) using Circulation software.

### Assessment of the degree of anterior descending coronary artery stenosis

**Fig. 1:**
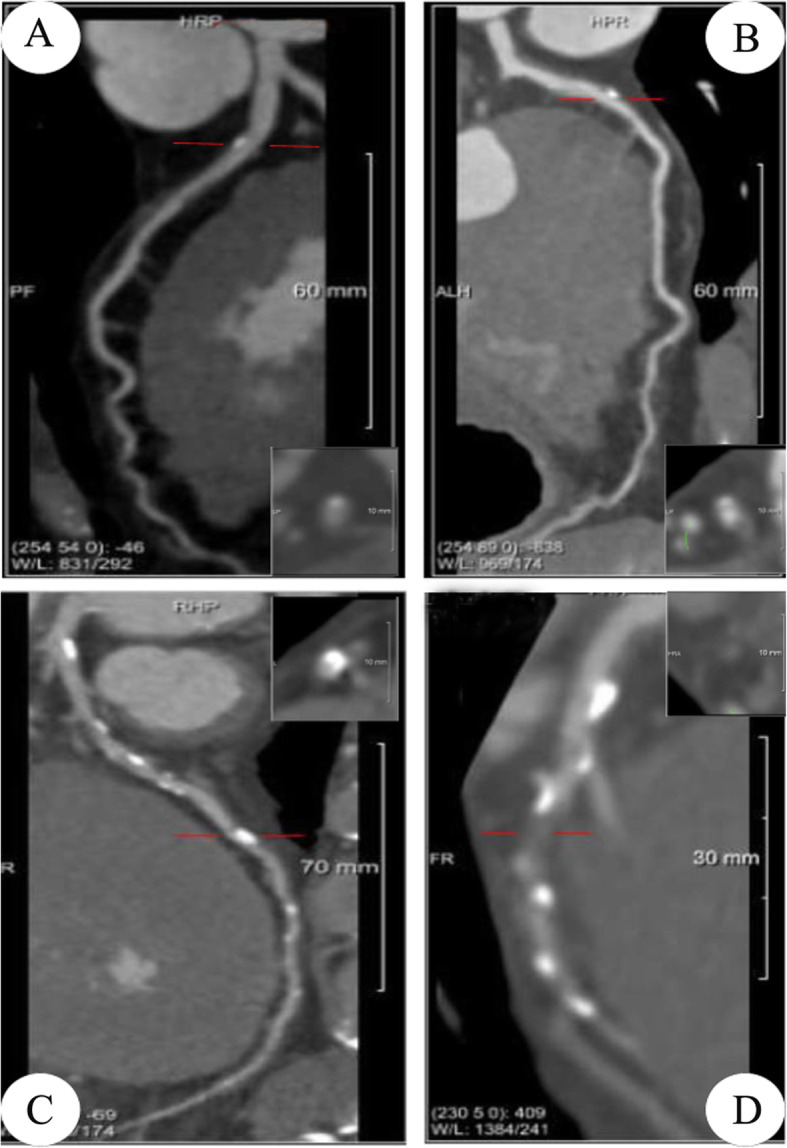
The percentage of left anterior descending coronary artery (LAD) stenosis defined by coronary CT angiography. The degrees of LAD stenosis were ≤ 25% (**a**), 26 to 49% (**b**), 50 to 74%(**c**) and ≥ 75% (**d**)

The percentage of LAD stenosis was defined by coronary CT angiography (CTA) (Fig. 1). Two radiologists with different degrees of experience in interpreting CTA images independently performed all qualitative image analyses. One was a senior radiologist (HS) with 25 years of experience in thoracic imaging; the other was a fellow (YC) with 5 years of experience in the interpretation of CT images. Both analyzed the Digital Imaging and Communications in Medicine (DICOM) images from the CT studies without access to the clinical findings. If their interpretations differed, the senior reader’s decision was accepted.

### Computed tomography feature tracking analysis

A commercial software package (Medis suite version 3.0 (Leiden, The Netherlands)) was used to analyze the original three-dimensional (3D) dataset package to generate two-dimensional (2D) cine loops of three long axis (LAX) slices (i.e., two-, three-, and four-chamber), three short axis (SAX) slices (i.e., basal, mid, and apical), and a SAX stack with a slice thickness of 0.75 mm and a reconstruction increment of 0.4 mm. Images were generated at a time resolution of 10 phases per cardiac cycle, increasing from 10% increments from the early systolic (0% cardiac cycle) to the end-diastolic (90% cardiac cycle). This was performed to ensure that the 2D cardiac CT reconstruction closely matched the anatomical location of the image.

Cardiac volumetric and functional parameters were quantified based on the manual delineation of the endocardial and epicardial borders using a stack of continuous SAX slice cine images (after excluding the papillary muscles from the myocardium). The left ventricular EDV, ESV and EF were obtained automatically.

**Fig. 2:**
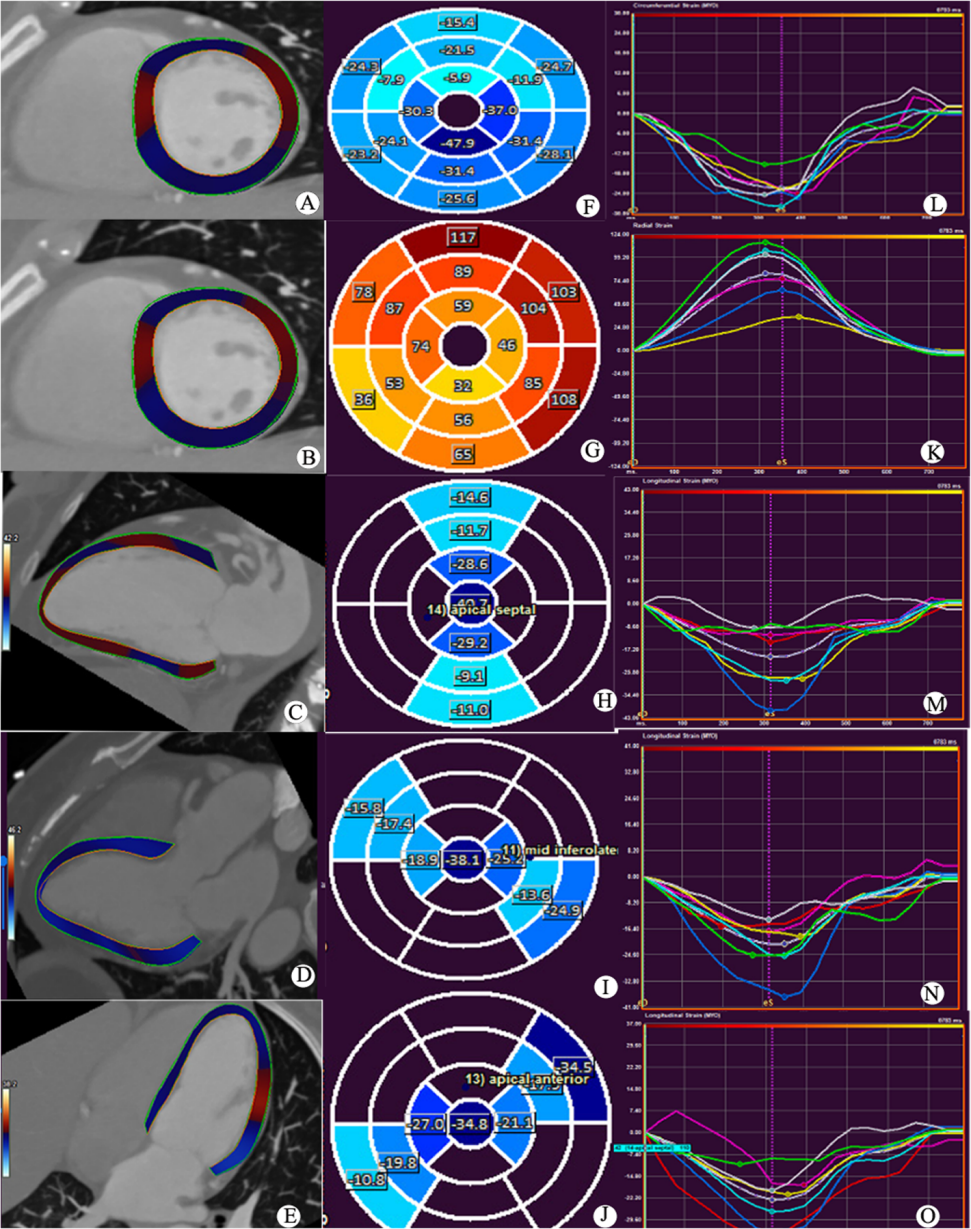
Diagram of the peak systolic strain analysis of the left ventricular myocardium in a healthy volunteer (a forty-seven-year-old female). Colored tissue-tracking maps of circumferential (**a**), radial (**b**), and longitudinal (**c**, **d**, **e**) strain analyses are shown on the left. The circumferential (**f**), radial (**g**), and longitudinal (**h**, **i**, **j**) strain values in a 16-segment model are displayed in the middle. Circumferential (**l**), radial (**k**), and longitudinal (**m**, **n**, **o**) strain-time curves in a cardiac cycle are shown on the right

**Fig. 3:**
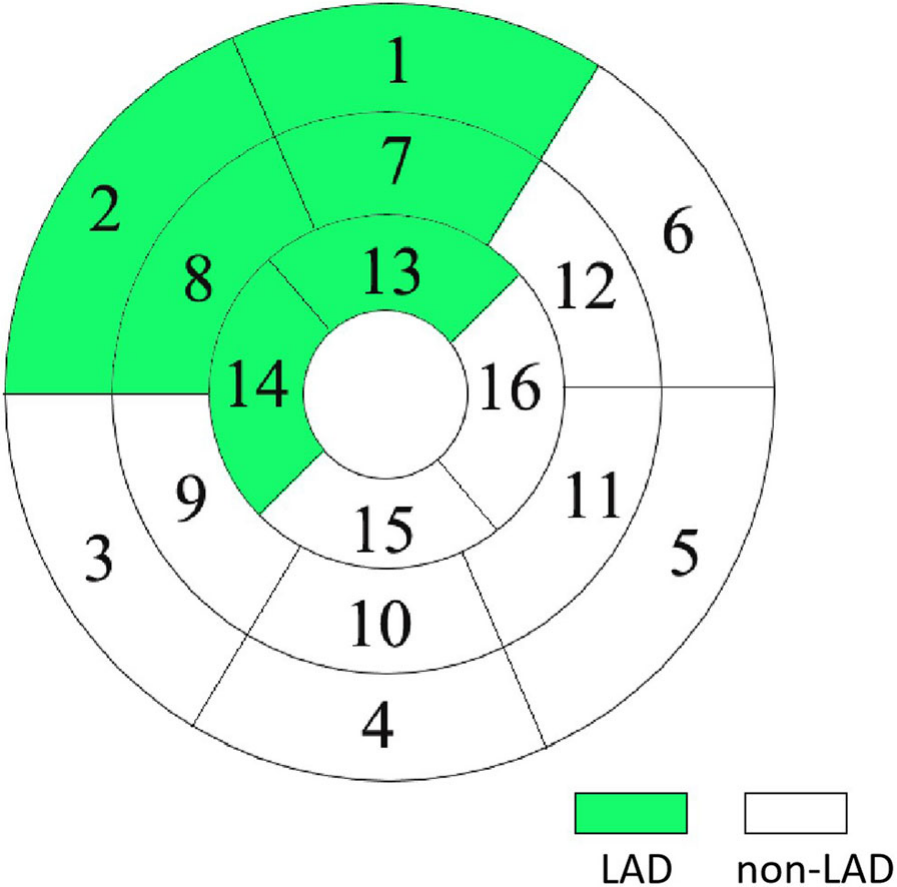
The involved segments as the territory supplied by the left anterior descending coronary artery (LAD) based on the American Heart Association (AHA) 16-segment model

Longitudinal strain (LS) was assessed by averaging the peak systolic strain values of 17 segments extracted from three LAX images (Fig. 2a), while circumferential strain (CS) and radial strain (RS) were acquired from three SAX images using a 16-segment model (Fig. 2b, c). To determine the reproducibility of the myocardial strain measurements, the same images from 15 randomly selected individuals were repeatedly measured by the same blinded observers. According to the protocol proposed by the American Heart Association (AHA), the coronary artery tree was divided into 16 segments [17]. Based on the AHA 16-segment model, we defined the basal and mid-anterior wall and anteroseptal segments as well as the apical anterior and septal segments as the territory supplied by the LAD (Fig. 3). All images were evaluated by two experienced cardiac radiologists who were blinded to the patients’ information (XH and JG). After separate evaluations, the radiologists discussed their findings and agreed on the final decision.

### Statistical analysis

For all continuous data, the normality of the distribution was checked using the Kolmogorov-Smirnov test. Normally and nonnormally distributed data and categorical variables are expressed as the means ± standard deviations and the medians (interquartile ranges) and frequencies (percentages), respectively. Independent sample Student’s *t* tests were used to compare two groups of normally distributed variables, and chi-square tests were used to compare categorical variables. Clinical characteristics and CT findings were compared among groups I-IV and the controls by one-way ANOVA. Normally distributed variables were analyzed by Pearson’s correlation analysis, and nonnormally distributed data were analyzed by the Spearman correlation analysis. Multiple linear regression analyses were performed to identify the determinants of myocardial LS in patients with LAD stenosis. All candidate variables (*p* < 0.2 on univariable linear regression without collinearity) were entered into the multiple stepwise regression model. Repeatability between observers was evaluated with 25 randomly selected patients with the Bland–Altman test. A *p* value < 0.05 (two-tailed) was considered statistically significant. Statistical analyses of all data were carried out using SPSS software (SPSS 21.0 for Windows, IBM, Chicago, IL, USA).

## Results

### Clinical characteristics of the study population

Among the 87 screened patients, eight were excluded because of poor-quality CT images, and three were excluded because of too many segments with poor tracking. Three of our patients had CAG results, and the percentages of LAD stenosis were 60, 75, and 90%. The remaining 73 patients were assessed by CTA. Then, the patients were divided into four groups according to the percentage of LAD stenosis: ≤25% was defined as group I (24 patients), 26 to 49% as group II (17 patients), 50 to 74% as group III (21 patients), and ≥ 75% as group IV (14 patients). Additionally, thirty-two sex- and age-matched healthy subjects were included as controls. During CTA examination, no significant difference in the average heart rate was found between the CAD groups and the controls (70.9 ± 12.7 bmp vs.69.7 ± 10.5 bmp, *p* = 0.986).

The demographics, clinical data and medical history of the subjects included in the present study are shown in Table 1. The mean age was similar between the patients and controls. Patients with LAD stenosis were predominantly male. This cohort had a high prevalence of hypertension, but there were no statistically significant differences between the groups with respect to hypertension, diabetes, smoking or alcohol consumption.

**Table 1:** Clinical characteristics of the study population

Variable	Group I (*n* = 24)	Group II (*n* = 17)	Group III (*n* = 21)	Group IV (*n* = 14)	Control (*n* = 32)	*P* values
Age (years)	56.7 ± 8	54.1 ± 12	62 ± 10	61 ± 13	56.7 ± 11	0.255
Male (n, %)	17 (70.8)	14 (82.4)	17 (81)	11 (78.6)	19 (57.6)	0.249
BMI (kg/m^2^)	24.9 ± 3	25.1 ± 5	23.9 ± 4	24.8 ± 3	24.8 ± 3	0.399
Smoking history (Yes/No)	9/15	5/12	7/14	7/7	10/22	0.730
Drink history (Yes/No)	8/16	4/13	8/13	5/9	7/25	0.707
Heart rate (beats/min)	70.1	71.1	69.4	71.0	69.7	0.986
Hypertension (n, %)	11 (45.8)	9 (52.9)	8 (38.1)	7 (50%)	0	0.204
SBP	136 ± 20	131 ± 18	141 ± 20	144 ± 23	126 ± 16	0.013*
DBP	88 ± 16	85 ± 15	88 ± 15	89 ± 12	79 ± 16	0.091
Diabetes mellitus (n, %)	4 (16.7)	1 (5.9)	5 (23.8)	2 (14.3)	0	0.386
Medication						
ACEIs (Yes/No)	9/15	7/10	9/12	9/5	–	–
Diuretics (Yes/No)	5/19	4/13	8/13	7/7	–	–
CCBs (Yes/No)	8/16	6/11	10/11	10/4	–	–
β-Blockers (Yes/No)	6/18	4/13	5/16	9/5	–	–
Statins (Yes/No)	2/22	3/14	3/19	6/8	–	–
Hematocrit (%)	139.5	140.7	133	138.3	132.2	–
Total cholesterol (mmol/L)	3.61	3.94	4.91	4.99	3.92	–
Triglycerides (mmol/L)	1.72	1.76	1.55	1.64	1.56	–
HDL-C (mmol/L)	1.1	1.58	2.61	0.952	1.31	–
LDL-C (mmol/L)	2.4	2.62	2.96	2.95	3.19	–

All data are expressed as the mean ± SD, percentage (number of participants), or median (interquartile range), as appropriate

*P < 0.05 between groups

*BMI* body mass index, *SBP* systolic blood pressure, *DBP* diastolic blood pressure, *LVH* left ventricular hypertrophy, *ACEIs* angiotensin-converting enzyme inhibitors, *ARBs* angiotensin II receptor blockers, *CCBs* channel blockers, *HDL-C* High density liptein cholesterol, *LDL-C* low density liptein cholesterol

### Volume and function of the left ventricle

No significant intergroup differences were found between groups I-IV and the controls in terms of the left ventricular ejection fraction (LVEF) (*p* = 0.586), end-diastolic volume (EDV) (*p* = 0.719) and end-systolic volume (ESV) (*p* = 0.257) (Table 1).

### Parameters of CT-FT-derived strain among the study groups

The longitudinal strain (LS) of the LAD territory was significantly reduced in groups I-IV compared with the controls (− 20.8% and − 18.6% and − 18.6% and − 17.0% vs 23.7%, respectively). The peak value of LS decreased as the stenosis degree of the LAD increased; specifically, the higher the degree of stenosis was, the lower the LS value. In addition, the circumferential strain (CS) of LAD territory was significantly reduced in groups III and IV compared with the controls and groups I and II (− 22.4% and − 22.1% vs − 25.4% and − 24.1% and − 25.3%, respectively), but the amplitudes of radial strain (RS) showed no significant differences among the five groups (Table 2).

**Table 2:** Cardiac CT parameter of the study population

Variable	Group I (*n* = 24)	Group II (*n* = 17)	Group III (*n* = 21)	Group IV (*n* = 14)	Control (*n* = 32)	*P* values
LVEF (%)	63.3 ± 9	63.6 ± 8	63.1 ± 9	59 ± 13	63.9 ± 7	0.586
LVEDV (ml)	90 ± 19	84.9 ± 30	82.5 ± 19	90.1 ± 17	87 ± 15	0.719
LVESV (ml)	32.8 ± 10	40.6 ± 18	30.9 ± 11	37.3 ± 17	32 ± 10	0.257
CS (%)						
LAD territory (%)	−24.1 ± 4	−25.3 ± 6	−22.4 ± 4^*,a^	− 22.1 ± 4^*,a,b^	− 25.4 ± 4	0.062
Non- LAD territory (%)	−25.6 ± 5	−24.4 ± 4	− 25.9 ± 4	− 22.9 ± 5	−27.4 ± 5	0.052
GCS (%)	−25 ± 4	−24.1 ± 4	− 24.7 ± 6	−22.4 ± 4	−26.6 ± 4	0.115
RS (%)						
LAD territory (%)	76.6 ± 25	79.1 ± 42	93.1 ± 56	63 ± 23	88 ± 38	0.186
Non- LAD territory (%)	90 ± 36	96.5 ± 80	74.9 ± 23	76.4 ± 26	84.3 ± 28	0.74
GRS (%)	85.1 ± 26	89.1 ± 55	81.4 ± 25	71.7 ± 23	85.3 ± 32	0.628
LS (%)						
LAD territory (%)	−20.8 ± 3	−18.6 ± 4^*,a^	−18.6 ± 3^*,a,b^	−17 ± 5^*,a,b,c^	−23.7 ± 3	<0.001
Non- LAD territory (%)	−22.0 ± 3	−21.9 ± 3	−21.9 ± 2	−20 ± 4	− 22.0 ± 4	0.488
GLS (%)	−20.1 ± 2	−20.3 ± 3^*^	− 20.5 ± 3^*,a^	−18.7 ± 4^*,a,b,c^	−22.9 ± 3	<0.001

All data are expressed as the mean ± SD, percentage (number of participants), or median (interquartile range), as appropriate

*P < 0.05 compared with normal controls

^a^*P* < 0.05 compared with groups I

^b^*P* < 0.05 compared with groups II

^c^*P* < 0.05 compared with groups III

*HR* heart rate, *LVEF* left ventricular ejection fraction. *LVEDV* left ventricular end-diastolic volume, *LVESV* left ventricular end-systolic volume, *RS* radial strain, *CS* circumferential strain, *LS* longitudinal strain, *GRS* global radial strain, *GCS* global circumferential strain, *GLS* global longitudinal strain

### Comparison of strain between LAD territory and non-LAD territory in groups I-IV

Compared with LAD non-territory, groups II-IV had significantly increased LS values in the LAD territory (− 18.6% vs − 21.9%, *p* = 0.07; − 18.6% vs − 21.9%, *p* = 0.024; − 17.5% vs − 20.1%, *p* = 0.032, respectively), which was not observed in the controls or group I. Group III had a significantly increased CS of the LAD territory compared with that of the non-LAD territory (− 22.4% vs − 25.9%, *p* = 0.03), which was not observed in other groups. However, no significant differences were found between the RS of LAD territory and that of non-LAD territory in the five groups (Table 3).

**Table 3:** Peak systolic strain of patients of between the LAD territory and non-LAD territory

Variable	Strain of LAD territory	Strain of non-LAD territory	*P* values
Group I			
CS	−24.1 ± 4	−25.6 ± 5	0.269
RS	76.6 ± 25	90 ± 36	0.133
LS	−20.8 ± 3	− 22.0 ± 3	0.085
Group II			
CS	−25.3 ± 6	−24.4 ± 4	0.625
RS	79.1 ± 42	96.5 ± 80	0.432
LS	−18.6 ± 4	−21.9 ± 3	0.007*
Group III			
CS	−22.4 ± 4	−25.9 ± 4	0.024*
RS	93.1 ± 56	74.9 ± 23	0.175
LS	−18.6 ± 3	−20.5 ± 3	0.030*
Group IV			
CS	−22.1 ± 4	−22.9 ± 5	0.856
RS	63 ± 23	76.4 ± 26	0.184
LS	−17 ± 5	−18.7 ± 4	0.032*
Control			
CS	−25.4 ± 4	−27.4 ± 5	0.203
RS	88 ± 38	84.3 ± 28	0.750
LS	−23.7 ± 3	−22.9 ± 3	0.431

All data are expressed as the mean ± SD, percentage (number of participants), or median (interquartile range), as appropriate

*P < 0.05 between groups

*LAD* left anterior descending, *RS* radial strain, *CS* circumferential strain, *LS* longitudinal strain

### Factors associated with myocardial strain of LAD territory in the patient group

**Fig. 4:**
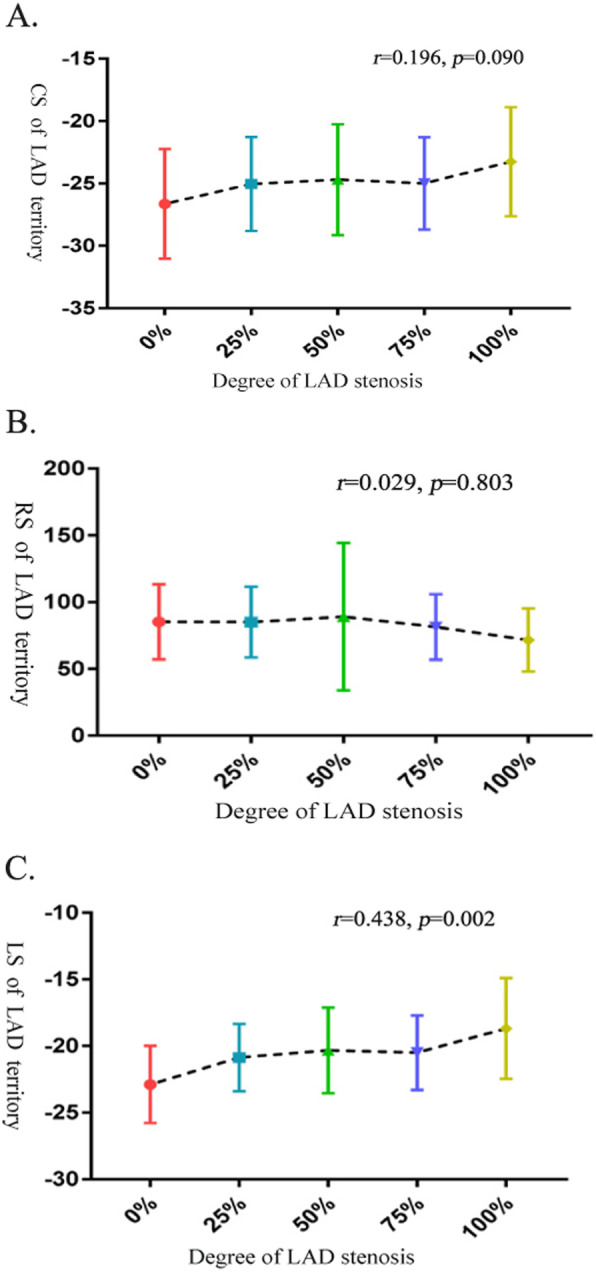
Correlations between stenosis of the left anterior descending coronary artery (LAD) and the amplitudes of circumferential strain (**a**), radial strain (**b**) longitudinal strain (**c**) and strain in the LAD territory

These results are shown in Table 4. In the patient group, the LS of the LAD territory was significantly correlated with the severity of LAD stenosis (*r* = 0.438, *p* = 0.002) (Fig. 4), angiotensin-converting enzyme inhibitor (ACEI) treatment (*r* = − 0.235, *p* = 0.041), the level of high-density lipoprotein cholesterol (HDL-C) (*r* = − 0.551, *p* = 0.008), and LVEF (*r* = − 0.315, *p* = 0.009). In addition, the CS of the LAD territory showed a significant negative correlation with the LVESV (*r* = − 0.324, *p* = 0.002) and LVEF (*r* = − 0.323, *p* = 0.005). The RS showed a negative significant correlation with the LVEDV (*r* = 0.304, *p*<0.008). However, no significant associations were observed between cardiac strain (CS, RS and LS) in the LAD territory and age, heart rate, hypertension, history of smoking, history of alcohol consumption, BMI, DM, or other medications.

**Table 4:** Univariate correlation coefficients for RS, CS and LS of LAD territory in patients with LAD stenosis

	CS (%)		RS (%)		LS (%)	
Variable	R value	*P* value	R value	*P* value	R value	*P* value
Age (years)	−0.062	0.597	− 0.013	0.912	− 0.07	0.548
Sex	0.104	0.371	− 0.023	0.845	0.242	0.073
BMI (kg/m^2^)	0.088	0.448	0.027	0.816	0.061	0.604
Hypertension	0.036	0.756	−0.126	0.276	−0.038	0.747
SBP (mmHg)	0.057	0.623	0.102	0.382	0.108	0.352
DBP (mmHg)	0.041	0.728	−0.081	0.488	0.042	0.721
DM	0.109	0.349	−0.136	0.241	− 0.020	0.864
History of smoking	−0.027	0.817	−0.14	0.227	−0.053	0.647
History of drinking	−0.035	0.764	−0.06	0.606	−0.108	0.351
HR	0.052	0.657	0.053	0.649	0.048	0.680
ACEIs (Yes/No)	−0.181	0.119	0.070	0.550	−0.235	0.041*
Diuretics (Yes/No)	−0.014	0.905	0.013	0.913	−0.184	0.112
CCBs (Yes/No)	−0.166	0.151	−0.041	0.241	−0.136	0.241
β-Blockers (Yes/No)	−0.020	0.864	−0.013	0.913	−0.110	0.345
Statins (Yes/No)	0.076	0.513	−0.011	0.922	−0.091	0.435
Hematocrit (%)	0.307	0.093	−0.067	0.736	−0.024	0.903
Total cholesterol (mmol/L)	0.211	0.322	0.262	0.216	−0.024	0.903
Triglycerides (mmol/L)	0.009	0.969	0.180	0.423	0.069	0.749
HDL-C (mmol/L)	0.115	0.610	0.219	0.328	−0.551	0.008*
LDL-C (mmol/L)	0.365	0.086	−0.125	0.579	−0.203	0.365
Degree of stenosis	0.196	0.090	−0.029	0.803	0.438	0.002*
LVEF (%)	−0.323	0.005*	0.115	0.324	−0.315	0.009*
LVEDV (ml)	0.092	0.427	−0.304	0.008*	0.083	0.474
LVESV (ml)	−0.324	0.002*	−0.1	0.388	−0.229	0.460

Correlation analysis are using Person correlation

*P < 0.05 between groups

*BMI* body mass index, *SBP* systolic blood pressure, *DBP* diastolic blood pressure, *LVH* left ventricular hypertrophy, *ACEIs* angiotensin-converting enzyme inhibitors, *CCBs* channel blockers. *HR* heart rate, *LVEF* left ventricular ejection fraction, *HDL-C* High density liptein cholesterol, *LDL-C* low density liptein cholesterol, *LVEDV* left ventricular end-diastolic volume, *LVESV* left ventricular end-systolic volume, *RS* radial strain, *CS* circumferential strain, *LS* longitudinal strain

### Intra- and interobserver reproducibility

The intra-class correlation coefficient (ICC) value in the intraobserver analysis was 0.973 for the percentage of LAD stenosis on CTA. The ICC of variability was good for the global strain values and the average segmental amplitude of strain in the LAD territory (Table 5).

**Table 5:** Intra- and interobserver reproducibility of CT-FT strain

Strain	Coefficient ofvariability (%)	ICC(95%CI)
Interobserver variability		
LAD CS	7.0	0.90 (0.86–0.93)
LAD RS	6.7	0.88 (0.79–0.96)
LAD LS	6.5	0.86 (0.84–0.89)
GCS	7.8	0.96 (0.93–1.00)
GRS	7.4	0.94 (0.92–1.00)
GLS	6.6	0.87 (0.85–0.93)
Intraobserver variability		
LAD CS	5.9	0.89 (0.84–0.94)
LAD RS	6.6	0.91 (0.86–0.96)
LAD LS	7.6	0.95 (0.90–0.98)
GCS	6.1	0.91 (0.88–0.93)
GRS	5.5	0.86 (0.83–0.91)
GLS	7.2	0.93 (0.90–0.95)

*RS* radial strain, *CS* circumferential strain, *LS* longitudinal strain, *GRS* global radial strain, *GCS* global circumferential strain, *GLS* global longitudinal strain

## Discussion

Decreased regional LV dysfunction in patients with critical stenosis of the LAD was detected by 2D speckle tracking echocardiogram (STE) by Iva et al. [18]. However, whether the decreased regional strain was related to different degrees of LAD stenosis is unclear. In the present study, CT-derived GLS and the regional LS of LAD territory were impaired in patients with LAD stenosis (≥25%) compared with the controls, although there were no significant differences in LV structural and functional parameters among the groups. In addition, a moderately negative correlation was found between the LS of LAD territory and the severity of LAD stenosis. This phenomenon indicated that myocardial ischemia exists in mild or moderate coronary stenosis and that a higher degree of stenosis leads to a lower LS value. A previous study indicated that a reduced GLS measured in nonobstructive CAD (stenosis ≥50%) was identified by 2D STE compared with stenosis<50% in patients in each major coronary artery [19]. Additionally, Iva et al. [18] showed decreased reginal LV dysfunction in patients with critical stenosis of the LAD on 2D STE. Using the same method, You et al. [20] found that LS was significantly reduced in myocardial segments supplied by coronary arteries with ≥75% stenosis compared with those subtended by coronary arteries with<75% stenosis and those in the control group. However, in a recent study, significant differences in 3D-STE parameters (GLS, GCS, GRS, area strain) were found between the severe and moderate stenosis groups and the slight and mild stenosis groups by Li et al. [21]. Meanwhile, Deng et al. [22] showed that patients with mild single vessel coronary artery stenosis had decreased area strain at the involved segments using four-dimensional echocardiography. The above differences in myocardial strain may be due to differences in demographic characteristics, clinical conditions, involved myocardial segments, or strain acquisition methods. The present study only included patients diagnosed with LAD stenosis (not stenosis of the left circumflex coronary artery or right coronary artery), which can better reflect the effect of the stenosis of different coronary arteries on segmental myocardial strain. Accordingly, the LV dysfunction response to coronary stenosis may begin early, possibly due to intermittent coronary spasm or transient decreases in coronary perfusion [23].

Interestingly, in our study, the LS of the LAD territory was significantly reduced in groups I-IV compared with the controls, while the CS of the LAD territory was reduced in the group with coronary stenosis ≥50%, and the RS and LVEF showed no significant differences among the groups. Meanwhile, compared with non-LAD territory, groups II-IV had a significantly increased LS in the LAD territory, but only group III had a significantly increased CS in the LAD territory, even though no significant differences were found between the RS of the LAD territory and the non-LAD territory in all groups. These results suggested that LS was more sensitive for detecting impaired myocardial function than CS or RS. A previous study indicated that as the degree of ischemia worsens, ischemia and necrosis spread across the wall from the endocardium to the epicardium in CAD patients [24]. Therefore, a possible explanation for this phenomenon could be that the endocardial myocardium is composed of longitudinally oriented myocardial fibers that are the most vulnerable to ischemic injury, which causes functional alterations at an early stage in this layer [25], and the LS in the endocardium was found to be better for the identification of significant stenosis of the LAD than that in the mid-myocardium or epicardium. In addition, the function of the subendocardial preliminary myocardial fibers is more easily deteriorated than the function of the radial peripheral fibers of the mid-wall. LS is an indicator of vertical function, and its ability to detect critical LAD size is better than that of LVEF and other strain parameters. Theoretically, in view of the above two reasons, it is not surprising that LS has a superior ability to detect LAD stenosis than LVEF and other strain parameters.

In addition, our findings showed that ACEI treatment was significantly correlated with greater segmental myocardial LS. This suggests a protective effect of ACEI on myocardial systolic function. Previous meta-analyses have shown that ACEIs have multiple potential cardioprotective effects: controlling blood pressure and reducing LV hypertrophy as well as potentially exerting anti-atherosclerotic effects [26, 27]. Furthermore, the mechanism of influencing the coronary artery is an improvement in endothelium-dependent vasodilatation by increasing the level of bradykinin, increasing the expression and activity of nitric oxide synthase and reducing the production of smooth muscle proliferators [28]. This is also in agreement with previous studies that indicated the amelioration of systolic function in diabetic patients through treatment with ACEI [29, 30]. Similarly, the structural repair of coronary arteries in hypertensive heart disease had been detected by long-term treatment with ACEI [31]. However, the effect of medication on myocardial LS in CAD patients still needs further research.

The present study has several limitations. First, this study was conducted in a single center with a small sample, limiting the generalizability of the conclusions. Second, the patients with CAD were not verified by CAG. An invasive examination was inappropriate for patients without an MI history. None of our patients had a history of myocardial infarction, and CTA was used to rule out coronary heart disease. Most patients had mild to moderate stenosis; therefore, no further coronary angiography was performed in most patients. Although CAG was not performed in all patients (only three patients underwent CAG), we used coronary CTA images to assess the degree of coronary stenosis. Third, the heart rates of some patients during CTA examination were higher than 60, which might have influenced the strain assessment. However, no significant difference in the average heart rate was found between the CAD groups and the controls. Fourth, coronary artery calcification scores were not calculated for these patients. Considering the radiation dose during examination, non-enhanced cardiac CT scans were not performed. Lastly, we did not obtain comparison data for cardiac strain on CT with 2D-strain echocardiographic or cardiac MR feature tracking in our subjects, which limits the impact of the findings. However, CT-derived strain has been shown to be closely correlated with both echocardiography and CMR in multiple studies [10–13].

## Conclusions

Using the CT-derived strain, our study showed that the subclinical regional LS was significantly reduced in LAD stenosis patients compared with the healthy controls and was inversely correlated with the severity of LAD stenosis. Moreover, ACEI treatment can ameliorate decreased regional LS. In the future, a comprehensive, large-scale study is warranted to further explore the effects of the degree of stenosis in the three coronary vessels and medication on LV systolic function in this population. CT-derived strain may be a novel biomarker to support early myocardial function assessments and therapeutic decision-making in ischemic cardiomyopathies or other related diseases.

## Data Availability

The datasets used and analyzed during the current study are available from the corresponding author upon reasonable request.

## Abbreviations

CAD: Coronary artery disease
LAD: Left anterior descending artery
MI: Myocardial infarction
CTA: Computed tomography angiography
CMR: Cardiac magnetic resonance
VR: Volume rendering
MPR: Multiplanar reconstruction
CPR: Curved planar reconstruction
MIP: Maximum intensity projection
SAX: Short axis
HR: Heart rate
LVEF: Left ventricular ejection fraction
LVEDV: Left ventricular end-diastolic volume
LVESV: Left ventricular end-systolic volume
RS: Radial strain
CS: Circumferential strain
LS: Longitudinal strain
GRS: Global radial strain
GCS: Global circumferential strain
GLS: Global longitudinal strain
ICC: Intra-class correlation coefficient
AHA: American Heart Association
HDL-C: High-density lipoprotein cholesterol
LDL-C: Low-density lipoprotein cholesterol
